# GBSC: graph-based sequence clustering method for similar short tandem repeats in protein sequences

**DOI:** 10.1093/bioinformatics/btag378

**Published:** 2026-06-13

**Authors:** Patryk Jarnot, Joanna Ziemska-Legiecka, Marcin Grynberg, Vasilis J Promponas, Aleksandra Gruca

**Affiliations:** Department of Computer Networks and Systems, Silesian University of Technology, Gliwice, 44-100, Poland; Institute of Biochemistry and Biophysics, Polish Academy of Sciences, Warsaw, 02-106, Poland; Institute of Biochemistry and Biophysics, Polish Academy of Sciences, Warsaw, 02-106, Poland; Bioinformatics Research Laboratory, Department of Biological Sciences, University of Cyprus, 1 University Avenue, Nicosia, 2109, Cyprus; Department of Computer Networks and Systems, Silesian University of Technology, Gliwice, 44-100, Poland

## Abstract

**Motivation:**

Short tandem repeats (STRs) are abundant in protein sequences and play important role in determining their structures and functions. Strikingly, the unusual compositional characteristics of tandem repeats break classical sequence analysis tools.

**Results:**

Here, we establish the first algorithm to effectively identify and cluster STRs: Graph-Based Sequence Clustering (GBSC) features linear time complexity, and clusters protein sequence fragments based on their STRs, while allowing for insertions and mutations and supporting the analysis of imperfect or cryptic repeats. Due to its computational efficacy, our algorithm can be used to systematically scan for patterns in large datasets. We compare our method both to state-of-the-art methods for identifying STRs in proteins and alternative clustering approaches. Unlike existing STR analysis methods, GBSC clusters repeat patterns rather than raw sequences, operating at the level of structural repeat identity, while tolerating biological variations and preventing erroneous merging of structurally and functionally distinct motifs. Whereas functional annotation is typically only available at the protein level, the functions of individual STRs and sequences of adjacent STRs remain largely unknown. On a challenging use case we here demonstrate and discuss how our method can be used to associate previously unannotated repetitive protein fragments with similar ones, allowing the transfer of annotation by similarity. For the first time, GBSC offers a tool that systematically extends this fundamental bioinformatics principle to low-complexity regions across large datasets.

**Availability and implementation:**

GBSC is available at GitHub https://github.com/patryk-jarnot/GBSC and https://doi.org/10.5281/zenodo.18965247. The data and scripts to reproduce the analysis are available at https://doi.org/10.5281/zenodo.16906653.

## 1 Introduction

Protein sequence clustering methods are fundamental in the discovery of protein properties, as they allow identify groups of potentially orthologous proteins by way of their sequence similarities and, based on the likely orthologous relationship, infer functions of so-far uncharacterized proteins from similar proteins that are better annotated. Existing methods fall into two broad categories: alignment-based and alignment-free approaches. Alignment-based tools such as CD-HIT (Li and Godzik 2006), UCLUST (Edgar 2010), and MMseqs2 (Steinegger and Söding 2017) group sequences by computing pairwise or representative-sequence similarity using local or global alignments, accepting sequences into a cluster when they meet a predefined identity threshold (Wei *et al.* 2023). While highly effective for redundancy reduction and homology-based grouping of full-length protein sequences (Suzek *et al.* 2015, Feldbauer *et al.* 2021), these methods assume positional independence of residues, which is a critical assumption known to be strongly violated in short tandem repeats (STRs). Consequently, they are fundamentally ill-suited for clustering such regions as STRs are inherently repetitive, low-complexity sequences whose alignment scores are dominated by repeat copy number and unit length rather than by the biological identity of the underlying pattern, causing structurally distinct STRs to be erroneously merged. Alignment-free approaches, including *k*-mer frequency profiling (Moeckel *et al.* 2024), feature vector methods based on physicochemical properties (He *et al.* 2017), and MinHash-based sketching (Abnousi *et al.* 2018), appear to offer a more flexible alternative, yet they inherit the same conceptual limitation: by reducing sequences to aggregate compositional or statistical descriptors, they discard the periodic structural information intrinsic to tandem repeats (TRs), rendering them equally unsuitable for pattern-based STR discrimination. More recently, representations derived from protein language models (PLMs) such as ESM-2 (Lin *et al.* 2023) and ProtTrans (Elnaggar *et al.* 2022) have been proposed as the basis for sequence clustering, with embeddings capturing contextual residue relationships (Pereira *et al.* 2025). However, PLM embeddings are trained primarily on globular, non-repetitive sequences, and while internal attention mechanisms can reliably detect periodic structure in ideal repeats, PLM performance has been shown to be low and unstable specifically for repeats containing insertions and deletions (Kesten-Pomeranz *et al.* 2026), a structural feature common in imperfect STRs, making PLM-based clustering unreliable for pattern-based STR discrimination.

As a result, functional low-complexity regions have to date largely been identified by dedicated tools for specific instances, typically exploiting instance-specific properties. For example, in an earlier study, we found that within the intrinsically disordered region of the delta subunit of bacterial RNA polymerase that includes a positively charged stretch followed by a negatively charged one. These stretches fold back on each other, compacting the overall structure while maintaining its flexibility, which seems to be essential for the subunit function. Searching for similar charge-patterned regions subsequently revealed other RNA-binding proteins (Kubáň *et al.* 2019). Similarly, to identify such regions, scientists used regular expressions for collagen-like repetitions (Teuscher *et al.* 2019) or sequence profiles to identify HEAT repeats (Kamel *et al.* 2021). While these approaches are already used for the automatic annotation of protein databases, low-complexity regions remain functionally uncharacterized (Sigrist *et al.* 2013, UniProt Consortium 2023).

Here, we introduce a complementary and generic approach to address this gap by providing a new method for investigating the likely functions of unannotated STRs, a specific class of low-complexity regions. These are known to evolve by replication slippage and often confer function in line with their biochemical properties (Lin and Kussell 2012). In contrast to existing methods for protein sequence clustering, our Graph-Based Sequence Clustering (GBSC) method introduces a conceptually distinct paradigm: it first identifies STR patterns using a graph-based approach and then clusters those patterns, rather than the raw sequences themselves. This two-stage, pattern-centric strategy operates at the level of structural repeat identity rather than sequence-level statistics. Crucially, it retains biological flexibility by tolerating insertions and point mutations within sequences sharing the same pattern, while overcoming a critical limitation of similarity-based methods that causes the erroneous grouping of structurally and functionally distinct STRs into the same cluster. To our knowledge, GBSC is the first STR clustering method to explicitly decouple pattern recognition from sequence similarity scoring, offering a more principled and biologically interpretable framework for repeat characterization.

Each GBSC cluster provides a collection of protein fragments with highly similar STRs for further analysis. We demonstrate on an illustrative use-case how this enables inference of the RNA/DNA-binding function of STRs that have no annotated function by combining protein-level functional annotation with STR similarity. This approach allows the transfer of protein-level annotation to higher-resolution knowledge about the STR itself, providing high-confidence candidates for experimental validation. By introducing the *s* measure for identifying the most functionally concise clusters, we provide, for the first time, a tool (https://github.com/agruca-polsl/gbsc_clusters_functional_analysis) that allows the systematic transfer of functional annotations by similarity to STRs in large datasets.

## 2 Materials and methods

In this section, we introduce the GBSC method and described its analysis. We began by outlining the identification process of GBSC, discussing its parameters, and providing an example of STR identification. We also illustrated how GBSC clusters STRs based on similarity. Then, we compared GBSC with other methods for clustering protein sequences. Finally, we enriched GBSC clusters with Gene Ontology annotations, validated their confidence, and analyzed the cluster containing RG repeats.

### 2.1 Identification

The identification and analysis of STRs requires a diverse toolkit, as these sequences often vary in purity and periodicity. Several computational tools have been developed to detect those patterns in protein sequences, each employing distinct algorithmic strategies. Among the most widely used detection tools, SEG identifies low-complexity segments based on local compositional complexity (Wootton and Federhen 1993), while fLPS detects compositionally biased regions using a statistical framework sensitive to amino acid composition (Harrison 2021). LCD-Composer extends this approach by combining amino acid composition and the linear dispersion of residues for flexible LCR identification (Cascarina and Ross 2022), and CAST offers an integrative compositional assessment of LCR structure (Promponas *et al.* 2000). Beyond compositional bias, repeat-oriented tools address sequence periodicity: GBA provides a graph-based method for repeat analysis (Li and Kahveci 2006), T-REKS employs a k-means clustering algorithm to identify periodic patterns (Jorda and Kajava 2009), XSTREAM uses heuristic pattern matching to detect repeat units across varying period lengths (Newman and Cooper 2007), and SIMPLE targets highly cryptic (non-tandem) repeats by flagging residues whose motif frequency exceeds that expected from randomized sequences (Albà *et al.* 2002). Given the diversity of these approaches—and the fact that no single tool captures the full spectrum of STR types—we provide a comparative analysis across multiple tools to demonstrate that the STR identification method implemented by the GBSC algorithm is comparable with other established methods, collectively covering a broad range of repeat types, thus validating its utility as a robust and comprehensive approach for STR characterization. This comparison is included in Supplementary Material 1, available as supplementary data at *Bioinformatics* online. Notably, a set of those methods for identifying compositionally biased and low-complexity regions, including the GBSC STR patterns identification algorithm, is available for intuitive use via the PlaToLoCo web server (Jarnot *et al.* 2020).

GBSC scans a sequence and constructs a directed graph of overlapping dimers to find repeating patterns. It marks fragments represented by cycles in the graph representation as repetitive regions. The method begins by dividing the sequence into dimers and iteratively processing them. For each dimer, it creates a node if it is absent in the graph. Each node has a lifetime variable initially set to 0. This lifetime variable is incremented by 1 during each iteration; however, if a node is revisited, its lifetime resets to 0. Nodes represented by two neighborly dimers in the sequence are connected by edges. The weights of these edges indicate the number of occurrences of adjacent dimers, initially set to 1 and increased each time a transition between the same dimers occurs in the same direction. If any disappearing edge has a weight exceeding the threshold, all edges in the current graph with weights above this threshold contribute to the graph representation of the STR. The boundaries of this STR in the sequence are determined by the first and last occurrence of nodes in the graph. Consequently, the method identifies STRs in protein fragments and provides a graph representation for them.

### 2.2 Identification—parameters

GBSC has several parameters that can be adjusted to identify STRs of interest. The *weight* threshold determines how many times an edge must be visited to mark the corresponding residues as repeatable. Therefore, it determines a minimal number of repetitions of a pattern. The *lifetime* threshold affects both a maximal repeat pattern length and a maximal insertion length between pattern occurrences. However, for the same parameter value, different insertion lengths are accepted for different lengths of the repeating pattern. For instance, with the lifetime threshold of 4, the maximal insertion length between QA runs is 2 (eg QAQAQAEDQAQAQA), and the maximal length of a repeating pattern without such gaps is 4. To better manage these aspects, we introduced additional parameters to manage a maximal gap (*max-gap-len*) and pattern lengths in STRs (*max-node-count*), separately. Another parameter, *min-node-count*, allows researchers to filter out homopolymers, as they are often well-studied. For increased flexibility in detecting mutations, the *include-orphan-nodes* parameter can be utilized. This parameter enables the identification of single-node repetitions, which can be particularly useful for finding motifs such as KKX, essential for ribosome biogenesis.

In this article, we utilize two distinct sets of parameters, referred to as *strict* and *relaxed*. The *strict* parameter set identifies exact repeats and those with low mutation content, while the *relaxed* set accepts more mutations in between repeats. The parameters for both sets are detailed in Table 1. In the subsequent sections, we refer to GBSC with *strict* and *relaxed* parameters as GBSC-strict and GBSC-relaxed.

**Table 1:** *Strict* and *relaxed* parameter sets.

	GBSC-strict	GBSC-relaxed
Weight	4	4
Lifetime	10	20
Max-gap-len	2	3
Max-node-count	6	6
Include-orphan-nodes	No	Yes

### 2.3 Identification—example

**Figure 1:**
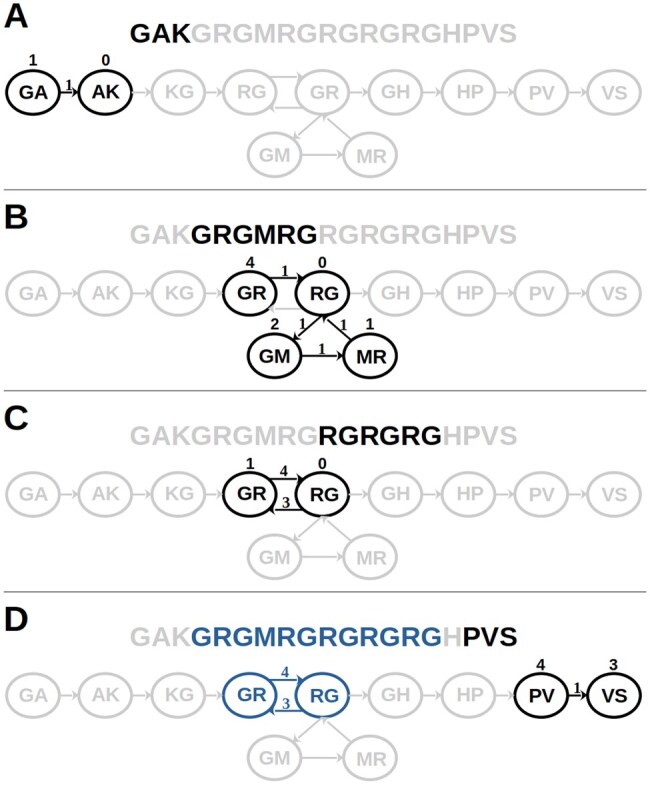
Example identification of RG repeats with a methionine insertion. (A) Shows the algorithm state after processing two *k*-mers. (B) The method handles insertion of a methionine. (C) It increases weights in edges already visited. (D) shows how GBSC marks a repetitive region and selects its graph representation after leaving it. In this example, the *lifetime* threshold was set to 5, and the *weight* threshold was set to 3.

Figure 1 illustrates how the GBSC method identifies RG repeats with a methionine insertion. In this example, we set the *lifetime* threshold to 5 and the *weight* threshold to 3. These repeats are derived from the coilin protein (P38432) and are part of the RG box, which is essential for interaction with SMN (Hebert *et al.* 2001). The example presents the RG repeats along with surrounding residues to provide a comprehensive view of how GBSC identifies STRs. Panel (A) shows the state after the second iteration, where the method has already created two nodes from the first two dimers of the sequence. The numbers above the nodes indicate their lifetime, while the numbers above the edges represent their weight. Panel (B) displays the step where the method approaches the RG repeats, encounters a single methionine insertion, and then returns to the RG repeats. Notably, nodes from previous steps that reached the lifetime threshold have disappeared. In the final step of this panel, the method revisits the existing RG node, resetting its lifetime. Panel (C) demonstrates how the edge weights increase as the method processes repetitions by revisiting them. In (D), scanning did not recognize any new repeats on its way. Overall, nodes with connected edges that have weights equal to or greater than the weight threshold are marked as repetitive, along with the associated sequence fragment.

### 2.4 Clustering

GBSC utilizes the graph representation of identified sequences to assign them into relevant clusters. Traditional clustering methods, such as Markov Cluster (MCL), are designed and optimized for full-length protein sequences, often requiring substantial computational power and memory to calculate and store statistical similarities between these sequences (Enright *et al.* 2002). Other tools attempt to mitigate this issue by minimizing the number of sequence comparisons. For instance, CD-HIT clusters sequences by comparing them only to representative sequences, assuming that remaining sequences are also similar to each other (Li and Godzik 2006). However, this assumption can fail when comparing adjacent STRs of different types, leading to the inappropriate mixing of STRs in a single cluster (Jarnot *et al.* 2022). In contrast, GBSC assigns STRs to clusters based on their graph representation established during the identification process. This method eliminates the need for a sequence comparison step, allowing sequences to be saved directly into their corresponding cluster files without occupying memory space. As a result, GBSC can efficiently manage large datasets, such as UniProtKB. Moreover, GBSC effectively addresses the challenge of adjacent STRs, where two or more STRs of different patterns are neighbors. The location of STRs within the sequence is known, enabling neighboring STRs to be assigned to clusters based on their types as well as to clusters representing their combinations. For example, if a poly-Q STR is adjacent to a QA run STR, they are assigned to three distinct clusters: a cluster with poly-Q sequences, a cluster with QA runs, and finally a cluster containing adjacent STRs of poly-Q and QA runs in the same order as they appeared in the sequence. This approach is unique compared to most protein sequence clustering methods, which typically assign each sequence to only a single cluster.

### 2.5 Alphabet reduction

In GBSC, sequences can be identified and clustered using a reduced alphabet. Certain amino acids in protein sequences share similar physicochemical properties, allowing them to be grouped into categories of similar residues. Researchers have defined several alphabet reductions, demonstrating their effectiveness in uncovering various aspects of similarity between proteins (Steinegger and Söding 2017, Liang *et al.* 2022). The GBSC method supports alphabet reduction passed as a parameter, which is applied when creating a graph representation of STRs. As a result, the identified and clustered sequences remain unchanged. This capability expands the range of STR repeats that GBSC can analyze. For instance, if we reduce the alphabet to three residual groups, which are glycine, proline, and others, then we can find collagen sequences with prolines at different positions in the triplet (GPX, GXP, and GPP).

### 2.6 Analysis—clustering

**Figure 2:**
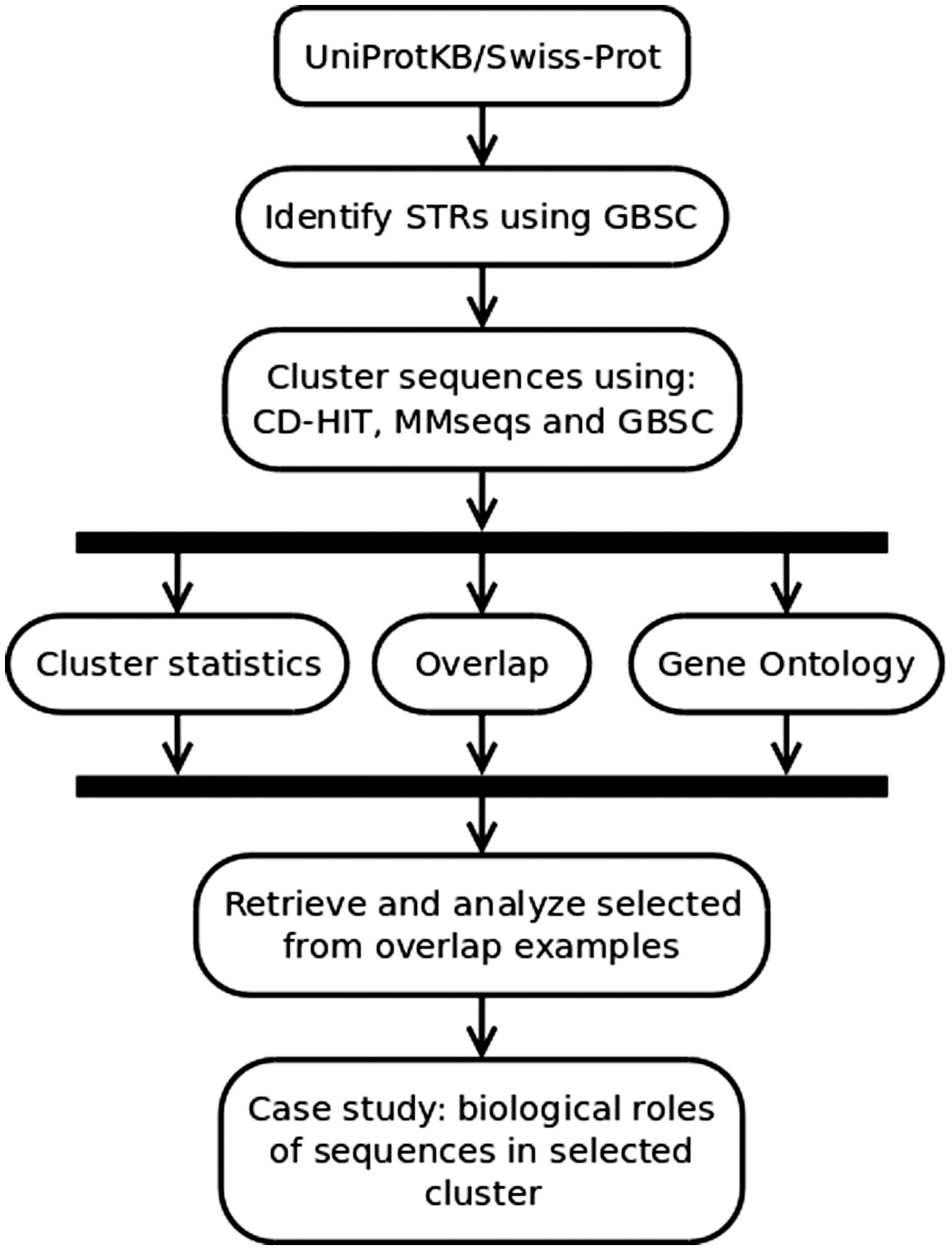
The workflow used to compare clustering methods. GBSC is used only to identify STRs in the UniProtKB/Swiss-Prot database. Next CD-HIT, MMseqs, and GBSC are used to create clusters of similar sequences which are then further analyzed. Clusters are firstly analyzed using an exploratory approach, then by examining the composition of selected examples and finally by examining at their biological roles.

GBSC is designed to cluster STRs according to similar, repeatable patterns, while state-of-the-art methods assess similarity between sequences using statistical approaches. To demonstrate the differences between these methodologies, we compared GBSC with CD-HIT v4.6 and MMseqs v15-6f452. The parameters used for STRs are detailed in Table 1. For all methods, we utilized STRs identified by GBSC in the UniProtKB/Swiss-Prot database version 2022_05 to focus solely on clustering solutions. We compared them quantitatively, providing statistics that describe the clusters and Venn diagrams showing the overlap between methods’ results. Since overlap cannot be directly derived from clusters, we paired all similar sequences by combining all possible combinations in each cluster. A pair of similar sequences was considered overlapping with other methods if both sequences belonged to the same cluster in those methods. From each section of the diagram, we selected representative examples to demonstrate the types of similarity detected between the STRs by each method. Additionally, we summarized the number of clusters containing adjacent STRs and the total number of such sequences to illustrate the impact of the GBSC solutions. Figure 2 presents the workflow of the clustering analysis.

### 2.7 Analysis—functional evaluation of clustering results with the *C* measure

**Figure 3:**
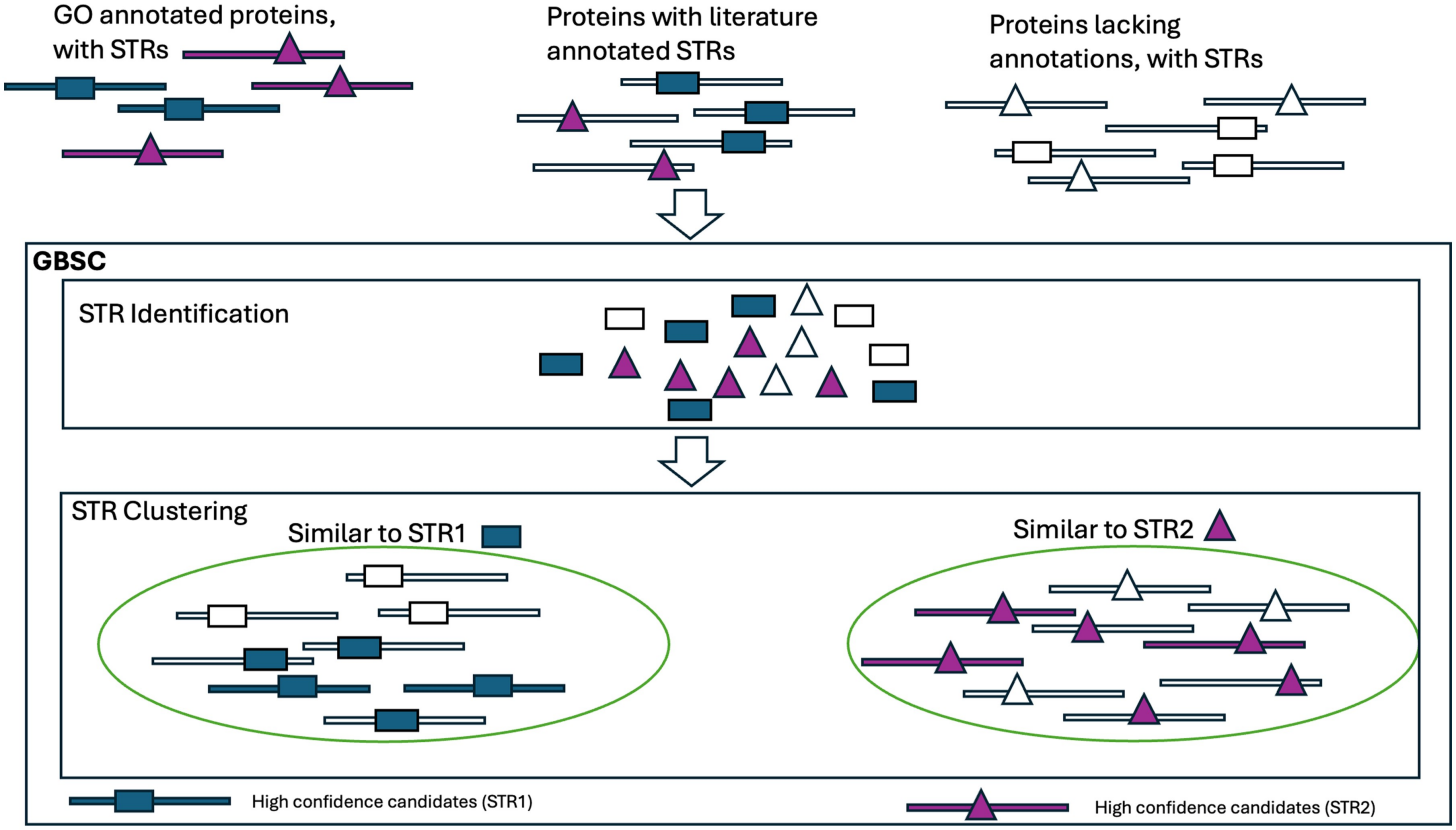
A workflow presenting the design of the Gene Ontology analysis. Proteins containing STRs and annotated with GO terms (functional annotation across the full protein sequence) that cluster with proteins with similar STRs, for which functional annotation is available from the literature, represent high-confidence candidates for experimental validation.

The GBSC method can be executed with various parameter settings, which can group functionally important sequences differently. Therefore, we are interested in finding a set of parameters that would allow the generation of the most functionally informative set of clusters as described by Gene Ontology annotations. Such a set should include a high number of clusters that contain STRs from functionally annotated protein sequences, as the known annotations allow us to hypothesize about the functions of other unannotated sequences within the same cluster by transferring protein-level annotation to higher-resolution knowledge about the STR itself (Fig. 3). The newly annotated STRs form high-confidence candidates for experimental validation. In addition, proteins with no annotated function in the same STR cluster provide further candidates for functional validation. To streamline the analysis, we designed the simple but informative *C* measure that allows us to find the best set of GBSC parameters that results in a high number of functionally annotated clusters. While evaluating the clustering results, the function takes into account the number of clusters annotated with at least one statistically significant GO term in the cluster, calculated with the hypergeometric test with the Benjamini–Hochberg multiple FDR correction procedure (Benjamini and Hochberg 1995) and normalized by the number of clusters.

In addition, we also provide the *s* measure that can be used to identify the most functionally concise clusters in the collection of clusters obtained using GBSC. This function takes into account the number of sequences in clusters that are annotated with the same, most frequent statistically significant GO term in the cluster. We provide the descriptions of the *C* measure and the *s* measure in the Supplementary Material 2, available as supplementary data at *Bioinformatics* online. For the *C* measure, we also provide a detailed analysis of how the different GBSC parameter values influence its values, the length of sequences within clusters, and the sizes of the clusters.

In order to identify an interesting case study, we analyzed the STRs in clusters obtained from GBSC analysis for all 216 parameter combinations, as described in Supplementary Material 2, available as supplementary data at *Bioinformatics* online. After a thorough analysis, one type of cluster with the repetitive pattern RG/GR caught our attention. It consistently appeared in the results with a statistically significant Gene Ontology term assigned and high percentage of proteins with assigned Gene Ontology terms. From these clusters, we selected the one with the highest value of the *s* measure, and we analyzed functions of its STRs.

## 3 Results

### 3.1 Statistics

As shown in Table 2, the clustering statistics for GBSC exhibited different trends compared to those of statistics-based methods when the parameters for STR identification were relaxed. MMseqs generated the highest number of clusters; however, many of these were singleton clusters, containing only one sequence. Interestingly, CD-HIT produced fewer clusters than GBSC for strict parameters but significantly more for relaxed parameters. All three methods resulted in an increase in singleton clusters when using relaxed GBSC identification parameters. Despite this, GBSC, on average, formed larger clusters with a greater standard deviation for relaxed parameters, while the other methods produced smaller clusters with lower standard deviations. The average number of sequences per cluster was highest for the CD-HIT method when using the strict set of parameters. However, for relaxed parameters, GBSC yielded the largest clusters on average. The standard deviations for the strict parameters were similar for GBSC and CD-HIT, in contrast to MMseqs. For relaxed parameters, the standard deviations for CD-HIT and MMseqs decreased significantly, while the standard deviation for GBSC increased slightly. In summary, for GBSC, all analyzed metric values increased with relaxation of the parameters, whereas for CD-HIT and MMseqs, the number of clusters increased, but the average number of sequences and the standard deviation decreased. The clustering parameters from the MMseqs and CD-HIT methods are available in the Supplementary Material 3, available as supplementary data at *Bioinformatics* online.

**Table 2:** Alignment based methods have different cluster tendencies than GBSC after relaxing parameters.^a^

Method	STR detection params.	Cluster count	Singleton cluster count	Mean seq. count per cluster	Std dev. of seq. count per cluster
GBSC	Strict	2913	1985	13.6	183.6
	Relaxed	6268	4078	15.3	189.1
MMseqs	Strict	9215	7774	4.2	52.0
	Relaxed	31 689	23 727	2.8	27.5
CD-HIT	Strict	1979	894	19.7	184.0
	Relaxed	14 158	7275	6.2	65.4

a The input dataset for the clustering methods was prepared by identifying STRs in UniProt/Swiss-Prot using two parameter sets: *strict* and *relaxed*.

### 3.2 Similar pairs overlap

Clusters formed by protein sequence similarity analysis methods should contain sequences that are similar to each other. Therefore, if we pick two randomly selected sequences from the same cluster, then these sequences should be similar. To examine the overlap between the results of different methods, we combined all pairs of sequences within the same cluster and created Venn diagrams to illustrate how the selected methods intersect.

Table 3 presents the number of similar sequence pairs generated by all three methods. GBSC groups sequences with similar repetitive patterns regardless of insertions and mismatches, whereas statistics-based methods take these factors into account. For strict parameters, GBSC detected 97.4% of all similar pairs identified by all methods, with CD-HIT following at 66.8% of the total similar pairs. However, after relaxing the parameters, GBSC discovered over twice as many similarities, while CD-HIT clustered approximately 25.7% of total similarities. This increase in the number of similar pairs in the GBSC results is primarily due to large clusters containing homopolymers with various mutations. MMseqs found the fewest similar pairs, accounting for 24.7% and 10.1% for strict and relaxed parameters, respectively. The total number of similar pairs is increased for relaxed parameters compared to strict by 234%, to which GBSC mostly contributes. Specifically, GBSC results increased by 228%, while the other methods found fewer similar pairs, indicating a divergence in the results among the methods. For the strict set of parameters, all methods identified over 50 million similar pairs, while this number exceeded 118 million for the relaxed set. Overall, GBSC found significantly more similar pairs after relaxing the parameters, whereas the other methods identified fewer. It is important to note here that in the case of GBSC, a single sequence motif can be assigned to multiple clusters, a feature not available in the other methods.

**Table 3:** GBSC found more pairs of similar sequences after relaxing parameters while rest of the methods less.

	Strict STRs	Relaxed STRs
GBSC	49 324 969 (97.4%)	112 687 201 (94.9%)
MMseqs	12 514 202 (24.7%)	12 029 273 (10.1%)
CD-HIT	33 861 449 (66.8%)	30 501 637 (25.7%)
Total	50 656 064 (100%)	118 715 870 (100%)

**Figure 4:**
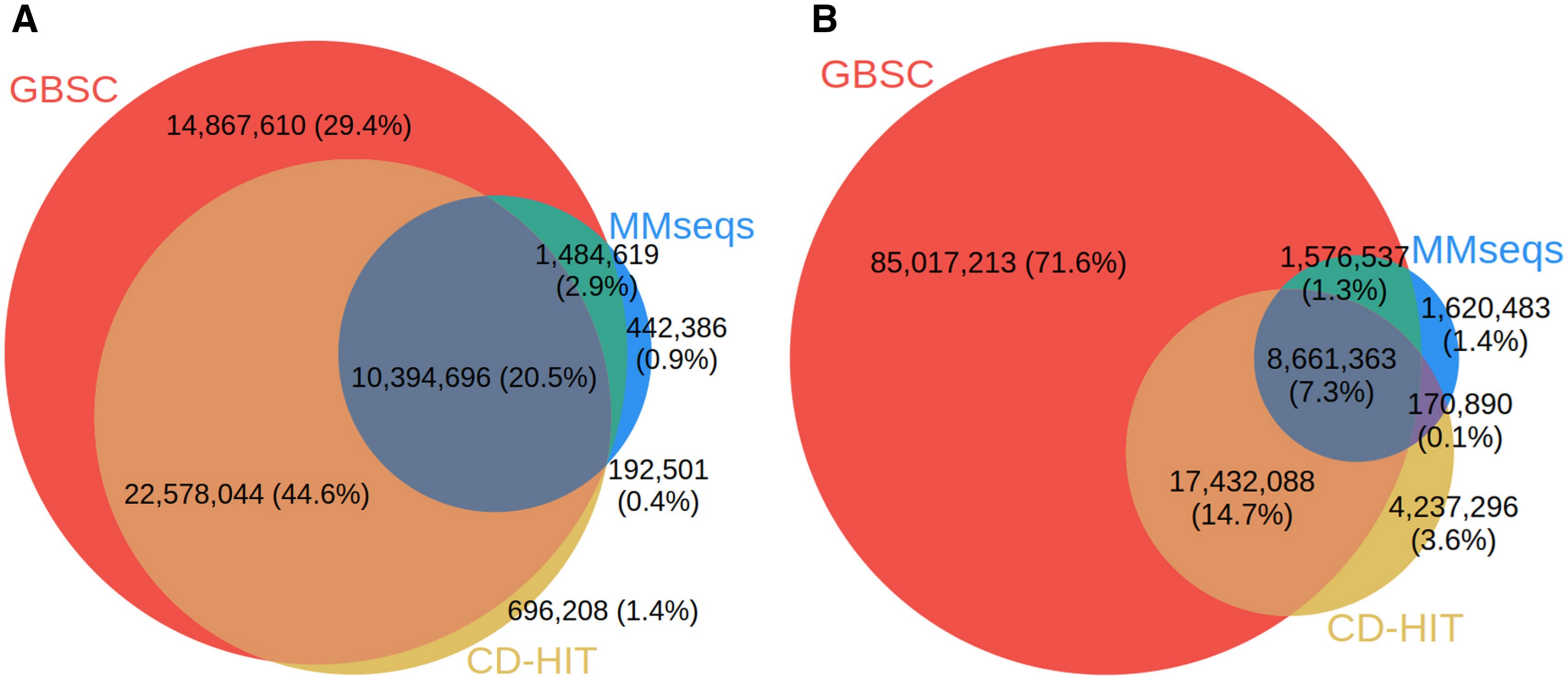
Venn diagrams showing overlap among GBSC, CD-HIT, and MMseqs when clustering STRs identified by GBSC. (A) shows results identified with GBSC-strict, while (B) shows results for GBSC-relaxed.

Figure 4 shows Venn diagrams for STRs identified by GBSC-strict and GBSC-relaxed. For strict parameters, CD-HIT and MMseqs shared most of the results with GBSC. The unique results we collected using these methods represented only a small part of the total results, which were 0.9% and 1.4%, respectively. CD-HIT, which generated more similar pairs than MMseqs, covered approximately 84.6% of the MMseqs results, which means that these methods aligned STRs differently, producing unique results in almost exact repeats identified by GBSC-strict. GBSC had a high overlap with the other methods, and 68% of all similar pairs were found by this method with CD-HIT, MMseqs, or both. 29.4% of all similar pairs were unique to GBSC, and only 2.6% were not detected by GBSC. For this set of parameters, we found 403 clusters containing 695 adjacent STRs, which partially characterize the red area of the diagram.

With relaxed parameters, the Venn diagram results showed significantly greater differences compared to those obtained with strict parameters. As previously noted, GBSC identified more similar pairs under relaxed conditions, while the other methods found fewer. Consequently, GBSC uncovered a much larger number of similar sequence pairs unique to this method. Specifically, 71.6% of the total similar pairs were identified solely by GBSC. This is justified by the fact that allowing more mutations within and between repetitions leads to more mismatches in alignments. GBSC shared 23.3% of similar pairs with the other methods, while 5.1% were found exclusively by the other methods. For this set of parameters, CD-HIT covered a smaller fraction of MMseqs results; that was 73.4%. The unique similar sequence pairs identified by CD-HIT and MMseqs represented 1.4% and 3.6% of all similar pairs, respectively, which was an increase compared to the strict parameter set. For relaxed parameters, we found 1067 clusters containing 8676 adjacent STRs.

### 3.3 Selected examples

**Figure 5:**
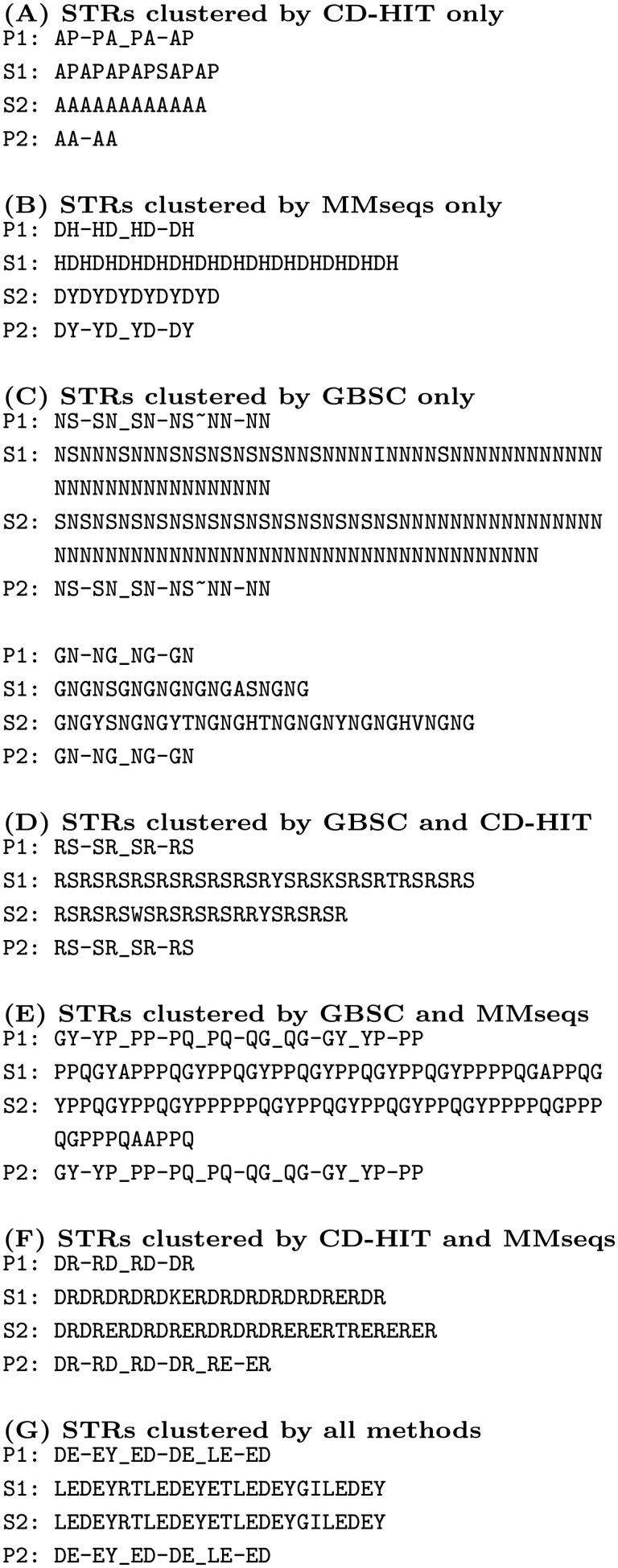
Selected examples that characterize all areas of the Venn diagram for strict parameters of GBSC. Each example is described by (P1) GBSC model assigned to the first sequence, (S1) sequence of the first region, (S2) sequence of the second region, and (P2) GBSC model assigned to the second sequence.

Figure 5 presents sample sequences for all regions of the Venn diagram shown in Fig. 4A. Figure 5A and B illustrate example pairs of potentially similar sequences clustered by CD-HIT and MMseqs. A closer examination of those cases reveals that both pairs were incorrectly clustered according to the repeating pattern of which they consist. The sequence pairs clustered by CD-HIT include an STR of alanine and proline alongside a poly-A fragment. Although both sequences are 50% identical, resulting in a high alignment score, this does not accurately reflect their similarity in terms of repeating patterns. A similar situation is observed with the two sequences in the MMseqs example, where the first sequence is an STR of the HD pattern, while the second consists of DY repeats.

Panel (C) shows two example sequence pairs containing repeating patterns according to GBSC. The first pair consists of two sequences made up of adjacent STRs, with the first part containing NS repeats and the second part being poly-N. These sequences differ in both length and the number of repeats of each type. The second example consists of sequences containing GN repeats. The first sequence contains a small number of mutations, while the second is highly degenerate and contain various mutations. Additionally, these two sequences also vary in length.

When GBSC overlaps with any of the other methods, the accuracy of STRs improves. Panels (D) and (E) illustrate examples where GBSC overlaps with CD-HIT and MMseqs, respectively. In the first case, where GBSC overlaps with CD-HIT, the sequences consist of varying numbers of SR runs. Both sequences contain several different mutations, but the predominance of exact repetitions enables both to be identified as similar by GBSC and the other methods. In the example where GBSC overlaps with MMseqs, the two sequences are composed of PPQGY, differing in the number of prolines that are degenerated at the C-terminal. These sequences contain similar repeats and can be well aligned.

The sequences presented in Panel (F) were interpreted differently by GBSC compared to CD-HIT and MMseqs. In the first sequence, GBSC identified only DR repeats, while in the second sequence, it recognized a combination of DR and ER repeats. As a result, GBSC assigned both sequences to different clusters. In contrast, CD-HIT and MMseqs classified both sequences as similar. Glutamic acid, which is abundant in the second sequence but rare in the first, was partially aligned and partially treated as mutations in the second sequence.

Panel (G) contains a pair of identical sequences. It is important to highlight that most pairs found at the intersection of all methods are either identical or contain only a small number of mutations.

### 3.4 Biological interpretation of selected cluster

In order to identify an interesting case study, we analyzed the STRs in clusters obtained from GBSC analysis. After a thorough analysis, one cluster caught our attention. In all of the 216 parameter combinations (see Supplementary Material 4, available as supplementary data at *Bioinformatics* online), this RG/GR cluster appeared in the results and had GO terms assigned. Detailed descriptions of proteins with assigned GO terms and motif RG/GR are provided in Supplementary Material 5, available as supplementary data at *Bioinformatics* online. We analyzed the group of 51 STRs from fungi, plant, and animal kingdoms characterized by the RG/GR motif. These are known RNA binding regions, named GAR domains, RGG boxes or RG domains (Burd and Dreyfuss 1994). The list of protein names obtained using the following parameters; *w *= 5, *g *= 3, *l *= 10, *x *= 4, is available in Supplementary Material 5, available as supplementary data at *Bioinformatics* online. Among those are well-known RNA binders, like U3 small nucleolar RNA-associated protein 25, interleukin enhancer-binding factor 3 or 40S ribosomal protein S2. There are also DNA binders, such as the transcription regulatory protein SNF2 and methyl-CpG-binding domain protein 2. All STRs from this cluster have very well established RNA/DNA binding capabilities. Supplementary Material 5, available as supplementary data at *Bioinformatics* online shows UniProt accession numbers and their STRs annotated as RNA/DNA binders either by sequence homology or by known functions according to the QuickGO database (Huntley *et al.* 2009). There are 18 protein STRs in this set that are known to bind DNA and 40 RNA binders. Out of 51 proteins, only 7 have no GO annotation. These are: A0A1D9BZF0 (germ cell nuclear acidic protein), A7TQN2 (SQS1), M9PBE2 (E3 ubiquitin-protein ligase Hakai), P12978 (Epstein-Barr nuclear antigen 2), Q3KSV2 (Epstein-Barr nuclear antigen 2), Q29EQ3 (PHD finger protein rhinoceros) and Q7YZH1 (PHD finger protein rhinoceros). This group of proteins has a common feature; all of them are known to bind proteins that bind RNA or DNA, but there is no evidence that they bind DNA/RNA in the RG/GR region (Hsieh *et al.* 1999, Pandit *et al.* 2009, Steele *et al.* 2009, Andersen *et al.* 2017, Glaser *et al.* 2017, Bawankar *et al.* 2021, Kelleher 2021, Ribeiro and Crossan 2023). Our results suggest that, apart from the ability to bind proteins, all those 7 proteins possess GR repeats known to bind nucleic acids, making them high-confidence candidates for further experimental validation.

## 4 Discussion

In this work, we introduced and analyzed a novel STR clustering method that utilizes the graph representation of STRs created during the identification process. This method focuses on clustering STRs based on their repetitive pattern, ignoring information about insertions and mutations between these repeats which makes it suitable for degenerate STRs. We compared this method with other approaches for both STR identification and protein sequence clustering. Subsequently, we conducted exploratory data analysis to investigate the results and examined selected cases in detail. Additionally, we performed Gene Ontology analysis to assess the functional composition of the clusters. Finally, we analyzed a specific cluster and discussed its biological relevance.

GBSC is able to cluster STRs composed of adjacent types, as it is aware of the locations of these motifs. Adjacent STRs can be critical for the functional and structural properties of proteins. For instance, a poly-P fragment inhibits *β*-Sheet structure in neighboring poly-Q fragment (Darnell *et al.* 2007). However, currently available tools for protein sequence clustering struggle to manage these adjacent STRs effectively (Jarnot *et al.* 2022). Methods like MMseqs and CD-HIT, which focus on whole protein sequences, skip information about STR locations, making it impossible for them to cluster adjacent STRs properly. In contrast, GBSC not only creates separate clusters for adjacent STRs but also appends their subtypes to their corresponding clusters.

GBSC clusters sequences based on repeating patterns while skipping information about mutations between them. Repeats in proteins can exhibit various mutations and insertions. A well-known example of this is collagen, which requires glycine at consistent intervals and permits nearly random residues in between (Naomi *et al.* 2021). However, statistics-based methods consider these insertions when calculating the final score, even allowing for changes in repetitive patterns (Jarnot 2024). As a result, fragments lacking relevant STRs may be aligned with a higher alignment score. By skipping these insertions when determining clusters for STRs, GBSC proves to be more suitable for such cases. A similar approach is employed by XSTREAM and T-REKS, where these repetitions serve as seeds for longer repeats (Newman and Cooper 2007, Jorda and Kajava 2009). However, these methods are designed only for the identification of STRs, not for clustering.

The sequence models produced by GBSC are interpretable, as GBSC tags identified and clustered sequences using a graph-based model of pattern that is interpretable and describes the sequences. Some tools for identifying regions with biased compositions lack descriptive information about the identified patterns which can be crucial for selecting the appropriate algorithm for a given task, even if a non-interpretable model has better performance (Linardatos *et al.* 2020). For example, T-REKS is a method suitable for exact and highly degenerate tandem repeats. However, it does not provide a consensus on what constitutes repeatability, making it challenging to understand which seeds were used during identification, and therefore it is unable to cluster TRs based on identified regions. Therefore, our method may be the optimal choice when the interpretability of similarity between STRs is a critical factor.

Finally, we have developed the *C* measure, an evaluation function to assess the functional composition of the GBSC clusters. We have also analyzed the influence of the GBSC parameters for the *C* measure values, identifying the optimal parameters for the Swiss-Prot database. The function uses annotation of Gene Ontology to protein sequences, a common approach in functional analysis of the gene or protein groups. However, in the case of STRs, such an approach can introduce errors, as those regions may not be associated with the Gene Ontology functions as they are usually assigned to the whole protein sequences. In addition to the quantitative analysis provided in Supplementary Material 5, we also presented a qualitative analysis of the cluster with an RG/GR motif that has been identified using the *C* measure. Our case analysis clearly shows previously missed functions of a set of proteins. We were able to hypothesize about it thanks to their presence in an RG/GR cluster with all other members being known to bind RNA or DNA.

In conclusion, it is well known that whole-protein-sequence-based clustering is not suitable for an analysis of low-complexity regions. More than half of all proteins, however, have low-complexity regions, with such regions even more prominent in “modern” proteins involved in development and the neuronal functions. To address this, we have developed GBSC, a new method to identify and cluster STRs, an important class of low-complexity regions. GBSC was developed with the purpose of analyzing in large protein datasets to functionally characterize STRs. The graph-based method supports the clustering of imperfect repeats as well as the clustering and interpretation of multiple adjacent STRs as single functional patterns. Notably, the constructed clusters comprise fragments with similar protein-level annotations. We have demonstrated the utility of this novel approach for inference the functions of the STRs themselves on a cluster of over 50 RG-repeat proteins, allowing us to newly link RNA/DNA-binding function with multiple STRs with high confidence.

## Supplementary Material

btag378_Supplementary_Data
