# Supplementary material for: GBSC: graph-based sequence clustering method for similar short tandem repeats in protein sequences
Source: Bioinformatics. 2026 Jun 13;42(7):btag378. doi: 10.1093/bioinformatics/btag378 (PMC13360283; doi:10.1093/bioinformatics/btag378)
Supplement: btag378_Supplementary_Data [file btag378_supplementary_data.zip › S01_identification_analysis.pdf]

Comparison of identification methods for motifs with biased compositions

Methods

Before clustering, GBSC must identify repeats, therefore we compared it with methods for identifying different types of non-standard amino acid composition to characterize its results. For comparison, we chose CAST v2.0, fLPS v2.0, GBA, SEG v1.0.0, LCD-Composer v1.0, T-REKS v1.3, XSTREAM v1.73 and SIMPLE with default parameters [1, 2, 3, 4, 5, 6, 7, 8]. We analyzed the results using an exploratory approach, showing the overlap between the selected methods by plotting the frequency of amino acids and their length distributions. As the input dataset, we used UniProtKB/Swiss-Prot version 2022_05, which contains manually curated records and has a low level of redundancy [9]. Additionally, we provide selected examples showing the differences in detail. Figure 1 presents the workflow of identification analysis.

Results

Length distribution

Compared to most methods, GBSC identified shorter fragments, while other methods merged adjacent STRs. The length distribution of the selected methods is shown in Figure 2. The majority of the fragments, that is about 87.9%, identified by GBSC-relaxed were in the range $6 \leq l \leq 20$, where l is the sequence length. Methods which found many regions in this range were XSTREAM, fLPS, GBA and partially SEG. However, even when other tools found longer fragments, they may contain multiple motifs of different types which are identified by GBSC separately. Such a method is SEG, which uses a complexity measure to identify motifs. An example of such a long motif containing multiple types of STRs is presented in Figure 3. This motif comes from the YRBM25_HUMAN RNA-binding protein (P49756) and consists of ER repeats that at some point changes into RD repeats. In this case, GBSC detected two short fragments, while SEG identified one long fragment. Another pair of methods include GBSC and SIMPLE. Both were designed to identify STRs, but they detected sequences of varying lengths with SIMPLE identifying notably longer sequences. This happens because a length of fragments identified by SIMPLE is close to its window size parameter. The lengths of motifs identified by CAST and fLPS ranged from several residues to hundreds of residues. These methods often detect long fragments that were identified by GBSC-relaxed as different types of STRs.

Amino acid frequencies

Figure 4 presents amino acid frequencies for selected methods and the UniProtKB/Swiss-Prot database. The average differences between each amino acid frequency of a method and the database were as follows: fLPS (0.13), CAST (0.014), SEG (0.014), XSTREAM (0.015), GBA (0.16), LCD-Composer (0.16), SIMPLE (0.024), GBSC-relaxed (0.028), GBSC-strict (0.035). The above list of methods is sorted starting with methods that return compositionally biased regions thought most imperfect repeats to the most ordered and shortest periodical repetitive patterns. These results suggest that sequences containing repeats have different amino acid preferences compared to irregular ones. We also see that STRs have different amino acid preferences than UniProt/Swiss-Prot

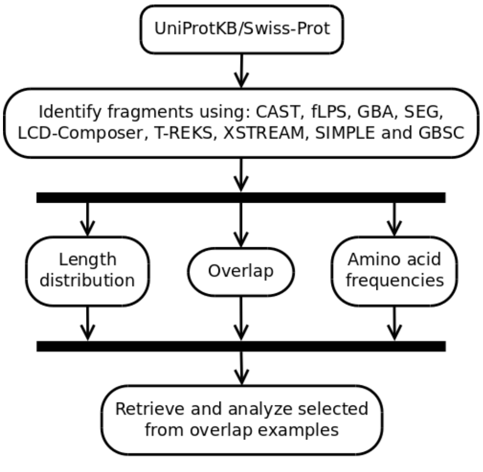

Figure 1: Workflow of the comparative identification analysis for diverse methods. Sequences are identified in UniProtKB/Swiss-Prot database by several methods. Then these sequences are analyzed using several approaches in an exploratory manner. Finally, selected examples are analyzed to show specificity of methods.

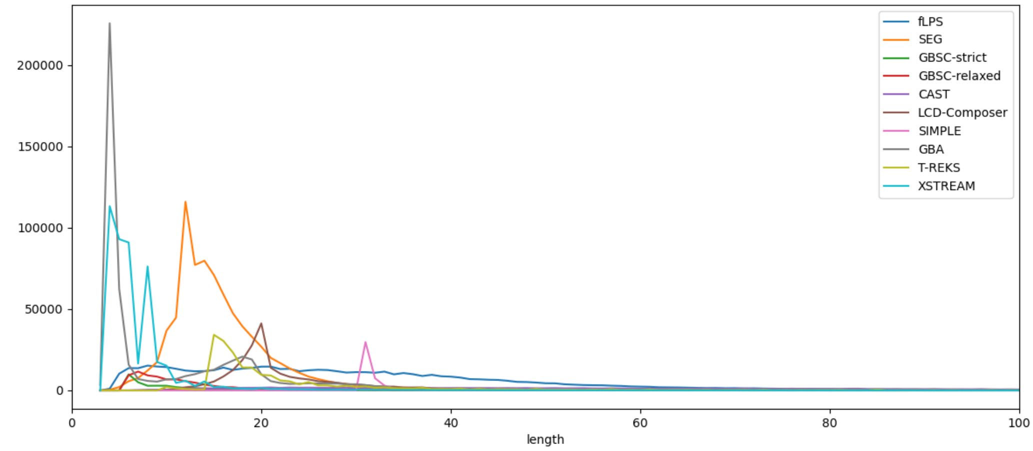

Figure 2: CBR identification methods are characterised by flat distribution of identified sequence lengths while rest of the methods have results cumulated near a single point. In GBSC, XSTREAM and GBA this point indicates shorter sequences than LCD-Composer, T-REKS and SIMPLE. This plot shows number of sequences (Y axis) by their lengths (X axis).

```
>sp|P49756|311|371|ER-RE~RD-DR
ERERERERERRERERERERERERERERinformationKEKERERERERDRDRDRTKERDRDRDRERDRDRDR
```

Figure 3: Adjacend STRs identified as separate STRs by GBSC and as a single motif by SEG. First part of sequence is RE repeats while the second part is RD repeats.

sequences. The five rarest residues in protein fragments containing short tandem repeats found by both GBSC variants and SIMPLE were tryptophan (W), phenylalanine (F), cysteine (C), isoleucine (I) and methionine (M). Interestingly, isoleucine occurs more often than by chance (¿0.05) in contrast to the GBSC-strict results where this frequency was below 0.01. On the other hand, the five most frequently occurring residues were asparagine (N), serine (S), glycine (G), glutamine (Q) and alanine (A) where asparagine and glutamine from this set occur less frequent than by chance in the database.

Amino acid overlap

The repeats identified by GBSC-relaxed largely overlapped with most methods for identifying non-standard residue composition, but interestingly differ from the STRs identified by SIMPLE. Table 1 shows the overlap of all fragments identified by the selected methods. GBSC overlapped mostly with CBR identification methods, which are CAST and fLPS. The fragments identified by these methods covered approximately 83% and 96% of the residues identified by GBSC-relaxed, respectively. Also, LCR identification methods largely overlapped with GBSC repeats. SEG and LCD-Composer covered these repetitions in 94% and 76%, respectively. (S)TR identification methods overlapped with GBSC in various ways. XSTREAM is a method that identified short fragments containing repetitions. It covered almost 72% of the GBSC-relaxed repeats. T-REKS, which found fragments of 15 or more residues, covered only 56% of the GBSC-relaxed repeats. The intersection between GBSC and SIMPLE was even smaller, accounting for less than 26%. Additionally, the length distribution of these methods showed even greater distinctiveness, as presented in Figure 2. It is worth noting that all methods identified a variable number of fragments of different lengths, hence the analysis of selected examples identified by each method adds value to the characterization of these methods.

Selected examples

For strict parameters, the total number of sequences identified by GBSC and missed by SIMPLE was 24 677. 91.4% of these fragments were homopolymers. On the other hand, SIMPLE found 2 861 fragments that were missed by GBSC. However, it is difficult to recognize a repeating pattern in most of these sequences. An example of a sequence identified only by SIMPLE is shown in Figure 5 panel **(A)**.

Sequences identified by T-REKS, excluding these identified by GBSC-strict, were in most cases highly degenerated. This even led to situations in which it was difficult to recognize a repeating pattern. An example of such a sequence is presented in Figure 5 panel **(B)**. This sequence consists of repeats that, according to theory, should start with an ST pattern followed by a variable number of trailing residues. The second example presents long tandem repeats, which GBSC avoided by design. The total number of TRs identified by T-REKS alone was 86 610.

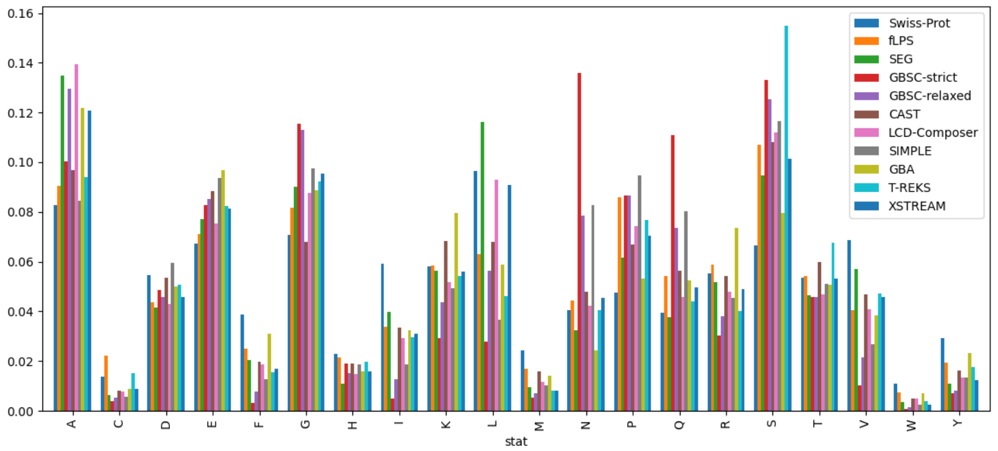

Figure 4: STRs have different amino acid preferences than other fragments with non-standard compositions and database sequences. Chart shows amino acid frequencies for fragments identified by selected methods and UniProtKB/Swiss-Prot database.

On the other hand, GBSC-strict identified 19 018 fragments missed by T-REKS. Most of them, about 98%, were short homopolymers, while the rest of the STRs consist of regular and irregular patterns. Examples are shown in Figure 5 panel **(C)**.

In comparison to T-REKS, XSTREAM found almost five times more sequences missed by GBSC-strict that was 409 975. These sequences were mainly short homopolymers or STRs consisting of up to 3 runs of repetitive patterns, which exceeded the GBSC threshold. Another type of fragments missed by GBSC-strict were Long Tandem Repeats (LTRs). The examples shown in Figure 5 panel **(D)** show sequences containing a short homopolymer and LTR identified by XSTREAM.

On the other hand, GBSC-strict found only 141 fragments missed by XSTREAM. Some of them contained irregular insertions between repeats, others were medium-sized STRs. However, XSTREAM had only a few of such sequences in its results, which lead to the hypothesis that these sequences were not recognized by XSTREAM rather due to an issue in its implementation. Example sequences are shown in Figure 5 panel **(E)**.

References

[1] Vasilis J Promponas, Anton J Enright, Sophia Tsoka, David P Kreil, Christophe Leroy, Stavros Hamodrakas, Chris Sander, and Christos A Ouzounis. Cast: an iterative algorithm for the complexity analysis of sequence tracts. *Bioinformatics*, 16(10):915–922, 2000.

[2] M Mar Albà, Roman A Laskowski, and John M Hancock. Detecting cryptically simple protein sequences using the simple algorithm. *Bioinformatics*, 18(5):672–678, 2002.

[3] Xuehui Li and Tamer Kahveci. A novel algorithm for identifying low-complexity regions in a protein sequence. *Bioinformatics*, 22(24):2980–2987, 2006.

[4] Sean M Cascarina and Eric D Ross. The lcd-composer webserver: high-specificity identification and functional analysis of low-complexity domains in proteins. *Bioinformatics*, 38(24):5446–5448, 2022.

[5] Aaron M Newman and James B Cooper. Xstream: a practical algorithm for identification and architecture modeling of tandem repeats in protein sequences. *BMC bioinformatics*, 8(1):1–19, 2007.

[6] Julien Jorda and Andrey V Kajava. T-reks: identification of tandem repeats in sequences with a k-means based algorithm. *Bioinformatics*, 25(20):2632–2638, 2009.

[7] Paul M Harrison. flps 2.0: rapid annotation of compositionally-biased regions in biological sequences. *PeerJ*, 9:e12363, 2021.

[8] John C Wootton and Scott Federhen. Statistics of local complexity in amino acid sequences and sequence databases. *Computers & chemistry*, 17(2):149–163, 1993.

[9] Uniprot: the universal protein knowledgebase in 2023. *Nucleic Acids Research*, 51(D1):D523–D531, 2023.

(A) Found in SIMPLE absent in GBSC

```
>sp|Q6ZPZ3|506|537|
PKPPPGVGLLPTPPRPPGPPAPTSPNGRPMQ
```

(B) Found in T-REKS absent in GBSC

```
>sp|O94278|471|485|
STDNSTLNEVSTEETS
>sp|Q17602|1019|1041|
EEPKSLETKVKEEPKPAVQTPVKE
```

(C) Found in GBSC absent in T-REKS

```
>sp|Q52KI8|275|290|RS-SR_SR-RS
RSRSRSKSRSRTRSRS
>sp|Q8NDV7|93|127|PQ-QQ_QP-PQ_QQ-QP_QQ-QQ
QQPQQQQQQQQPQQQQPQQQPQPQPQQQQPQQQPQ
```

(D) Found in XSTREAM absent in GBSC

```
>sp|Q57XV5|438|441|
RRRR
>sp|P44269|49|93|
AEGKCGEGKCGADKAKSAEGKCGEGKCGADKAKSAEGKCGEGKCG
```

(E) Found in GBSC absent in XSTREAM

```
>sp|Q7SIB2|802|823|GP-PP_PP-PG
GPPGAMGPPGGQGPPGSSGPPG
>sp|Q6UR67|209|220|HS-SH
HSHSHDSHSHSH
```

Figure 5: Selected examples which characterize GBSC fragments in comparison to rest of the methods for (S)TR identification.

Overlap results of identification methods

	cast	flps	gba	gbsc (relaxed)	gbsc (strict)	lcd-composer	seg	simple	treks	xstream
cast	18,129,024	7,396,409	1,263,552	947,618	471,819	3,565,831	5,066,023	628,406	1,815,399	1,919,929
flps	7,396,409	13,812,877	1,436,352	1,093,755	499,016	4,228,890	5,829,852	559,510	1,775,806	2,071,087
gba	1,263,552	1,436,352	4,825,550	305,448	161,228	806,489	1,390,621	156,401	375,255	638,689
gbsc-relaxed	947,618	1,093,755	305,448	1,144,582	494,849	869,144	1,081,459	293,248	636,898	818,611
gbsc-strict	471,819	499,016	161,228	494,849	501,157	391,222	485,854	219,679	333,853	485,459
lcd-composer	3,565,831	4,228,890	806,489	869,144	391,222	5,372,664	3,746,029	500,984	1,214,186	1,280,495
seg	5,066,023	5,829,852	1,390,621	1,081,459	485,854	3,746,029	12,052,107	527,936	1,656,244	2,171,070
simple	628,406	559,510	156,401	293,248	219,679	500,984	527,936	733,462	339,008	355,680
treks	1,815,399	1,775,806	375,255	636,898	333,853	1,214,186	1,656,244	339,008	2,995,775	1,408,019
xstream	1,919,929	2,071,087	638,689	818,611	485,459	1,280,495	2,171,070	355,680	1,408,019	4,000,128

Table 1. A high overlap GBSC has with CAST, fLPS, SEG and XSTREAM. Low overlap it has with SIMPLE and GBA. The table presents the number of identified residues overlapping between methods. The diagonal indices the total number of residues identified by each method.
