# Supplementary material for: GBSC: graph-based sequence clustering method for similar short tandem repeats in protein sequences
Source: Bioinformatics. 2026 Jun 13;42(7):btag378. doi: 10.1093/bioinformatics/btag378 (PMC13360283; doi:10.1093/bioinformatics/btag378)
Supplement: btag378_Supplementary_Data [file btag378_supplementary_data.zip › S02_functional_evaluation_of_clusters.pdf]

Functional evaluation of GBSC clusters with the *C* and *s* measures

Method

For the problem of functional similarity analysis of STRs we are interested in finding a set of parameters that would allow the generation of the most functionally informative set of clusters. Such a set should include a high number of clusters that contain STRs from functionally annotated protein sequences, as the known annotations allow us to hypothesize about the functions of other unannotated sequences within the same cluster. For that reason, we designed a simple but informative *C* measure (Eq. S1) that can be used to find the best set of GBSC parameters that results in a high number of functionally annotated clusters.

$C = \frac{number\_of\_clusters\_with\_at\_least\_one\_significant\_GO}{number\_of\_clusters}$, where: (Eq .S1)

- $number\_of\_clusters\_with\_at\_least\_one\_significant\_GO$ is the number of clusters that contain at least one significant GO term, excluding clusters with only one sequence.
- $number\_of\_clusters$ is the number of clusters generated.

To determine which GO terms are significantly enriched in clusters we are using hypergeometric tests with Benjamini - Hochberg FDR multiple correction procedure.

While having a set of clusters generated with a particular set of GBSC parameters, one may be also interested in assessing the individual clusters. Therefore, we propose another measure that can be used to identify the most functionally concise clusters within the collection of clusters generated by the GBSC method. The s measure (Eq. S2) takes into account the number of occurrences of the most frequent significant GO term in the cluster, that is the GO term assigned to the highest number of sequences within that cluster. Note that if there are multiple STRs from the same protein sequence, we count them as a single occurrence. Finally this number is normalized by the number of proteins in a single cluster as follows:

$$s = \frac{cluster\_sign\_GO}{cluster\_size} \qquad \text{(Eq .S2)}$$

where:

- $cluster\_sign\_GO$ is a number of sequences with a significant GO term annotating the highest number of sequences in the cluster.
- $cluster\_size$ is the number of sequences in the cluster.

The above equation can assess the consistency of clusters generated for a given GBSC parameter setting. The scripts to run this analysis for custom clusters are available on our github repository.

Below we present the analysis of different GBSC parameter settings and their influence on the *C* measure values. Protein sequences were downloaded from the UniProtKB/Swiss-Prot database (ver 2023_03) in the FASTA format. As a source of GO terms, we used the QuickGo database. We excluded GO terms assigned with evidence *inferred from Electronic Annotation* (IEA). In the analysis we excluded GO terms with the depth (length of the longest path from the root of the Directed Acyclic Graph to the GO term) lower or equal to four. We also disregarded clusters containing only a single sequence. If a cluster contains multiple STRs coming from the same sequence, we count the annotation for that sequence only once.

To understand how the values of the *C* measure depends on GBSC parameters, we calculated it for the following ranges of parameter values:
w - weight = 3, 4, 5
g - max-gap-len = 1, 2, 3, 4,
l - lifetime = 10, 12, 14, 16, 18, 20
x - max-node-count = 4, 6, 8
The parameter *m* - *include-orphan-nodes* was not set

The number of combinations of the values above for GBSC parameters was 216. For each combination of the parameters we have run the GBSC method and calculated the value of the *C* measure.

Results

The results of the evaluation function (*C* measure) of GBSC clusters were compared for different sets of parameters. Depending on the parameter settings the number of obtained clusters varied from 1192 (for *w*=5, *g*=1, *l* =10, *x*=4) to 10,408 (for *w*=3, *g*=4, *l* =20, *x*=8)

The percentage of singleton clusters, which contain only a single sequence, remains relatively stable regardless of the parameters (see Table 1 in Supplementary Material S2.1), and were excluded from the presented results. The number of clusters with only one protein has been stable between 65% and 75% for analyzed sets of parameters.

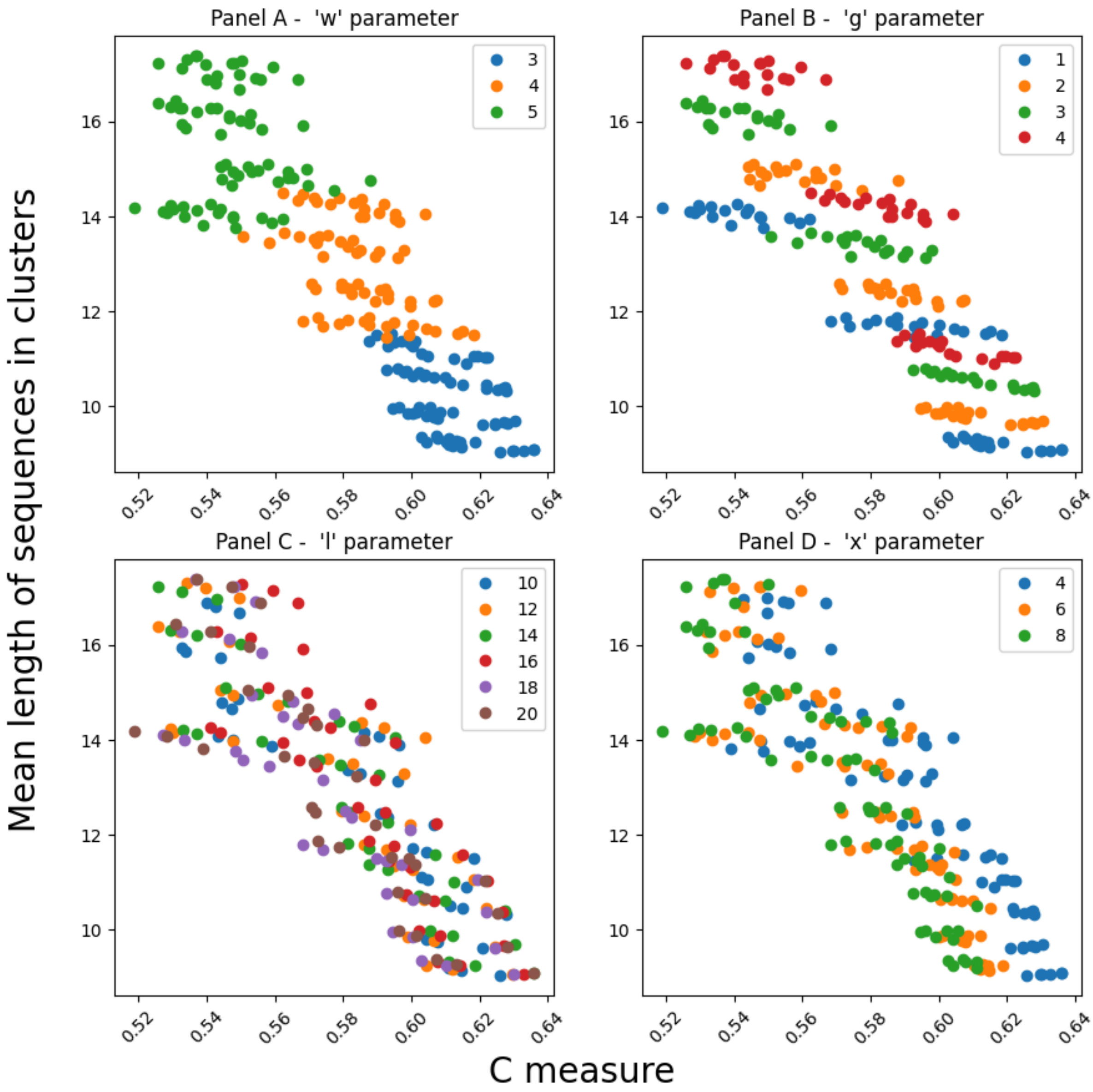

Fig S1. The relationship between the values of the GBSC parameters, the value of the C measure, and the mean length of sequences in clusters. A single dot on the plots represents the clustering results for a unique combination of the parameter values. All subplots present the same clustering results; different colors denote different parameter values. For some of the parameters, we can notice the groups of colors on the charts, which show how the presented parameter values influence the length of STR sequences in clusters, which is then reflected by the *C* measure value. We observe that the shorter the sequences in the clusters, the higher the values of the *C* measure.

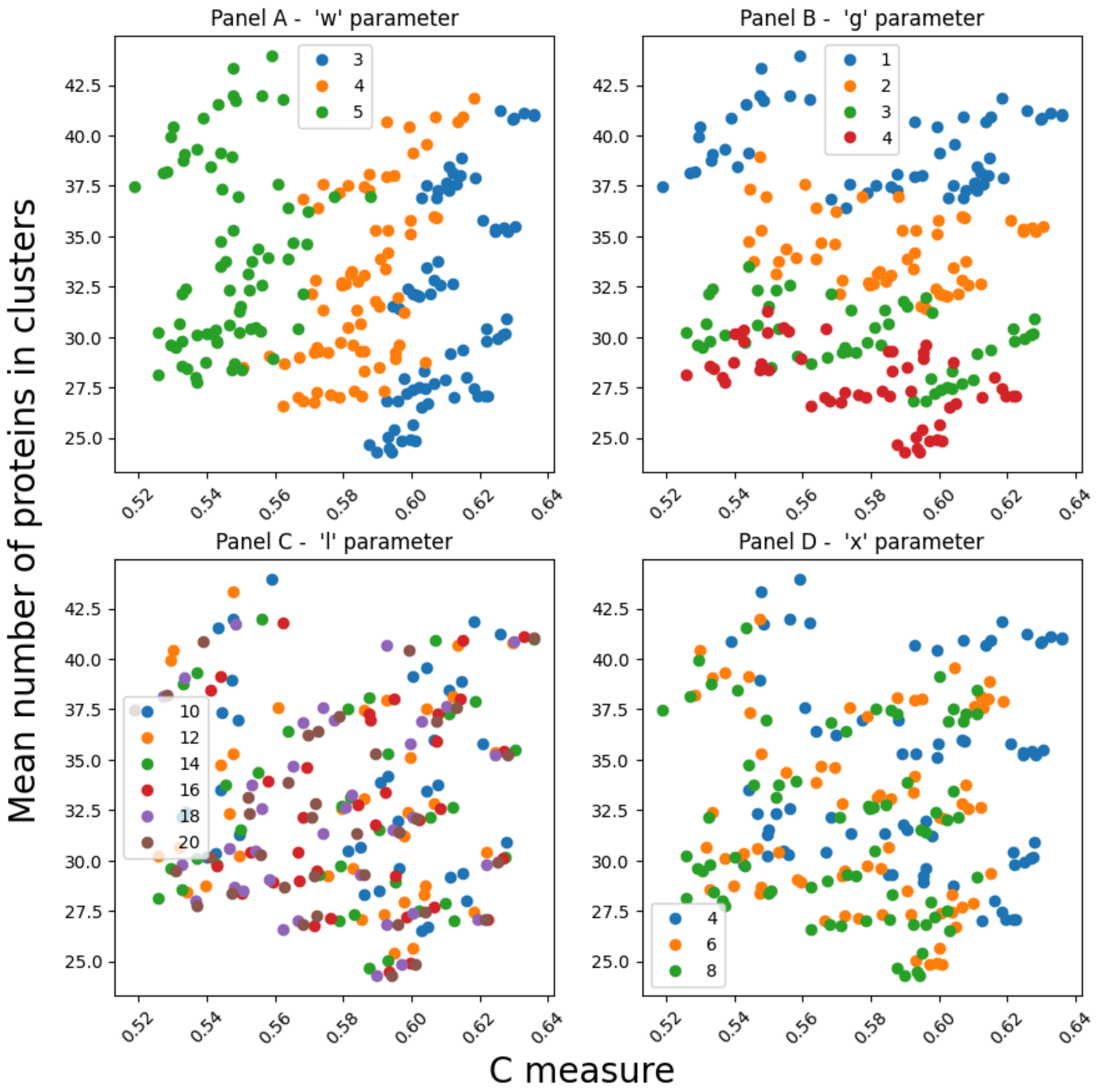

Fig S2. The relationship between the values of the GBSC parameters, the value of the *C* measure, and the sizes of clusters. A single dot on the plots represents the clustering for a unique combination of the parameter values. All subplots present the same clustering results; different colors denote different parameter values. We can notice the groups of colors on the charts, which show how the presented parameter values influence the size of the cluster. However, we do not notice the dependencies between cluster size and the values of the *C* measure.

By analyzing the results presented in Figure S1 we noticed the relationship between the mean length of sequences in clusters and the values of the *C* measure. The shorter the sequences are, the higher the values of the *C* measure. The length of the STRs detected by GBSC were mostly influenced by the values of the parameter *w*, and indeed, we observe a very clear separation of colour groups for this parameter (Fig S1, panel A). According to the definition, the higher the values of the *w* parameter, the longer repeats are required for the STR to be identified. Therefore, in the case of the higher values of this parameter, some of

the sequences with known functional annotation, but having shorter STRs, might not be detected, resulting in clusters lacking significantly enriched GO terms.

When analysing the values of the parameter *g* (panels B in Fig. S1 and S2), we also see the influence of its value on the mean length of STRs in the cluster (Fig. S1, panel A). However, this dependence is visible only within the groups first defined by the parameter *w* and is not related to the value of the C measure. In Fig. S2, on panel B, we can further notice that its values influence the size of the clusters. The *g* parameter defines the length of gaps between STRs and as its value becomes more relaxed, the size of the clusters decreases, as the two perfect STRs next to each other with a gap in between, will form a single STR.

The values of the parameter *x* (panels D in Fig. S1 and S2) influence the values of the *C* measure, but again within the clusters first defined by the parameters *w* and *g*. The lower its value within these clusters, the higher is the value of the C measure. The *x* parameter defines the maximum length of repeats.

Finally, the values of the *l* parameter (panels C in Fig. S1 and S2) reveals no dependencies related to length of sequences in clusters, cluster sizes and the values of the *C* measure. However, values of this parameter are directly related to values of parameters *g* and *x*. If the values of the lifetime parameters are too low, the longer repeating patterns will not be detected.

In Figure S3 we present the histograms of the *s* measure values (Eq. S1) for the GBSC clustering results that we obtained for the lowest and the highest *C* value for the analyzed dataset. The highest value of the *C* measure (*C* = 0.635983) has been obtained for the following parameters: *w* = 3, *g* = 1, *l* = 14, and *x* = 4 for 4,141 clusters. The lowest value of the *C* measure (*C* = 0.518760) has been obtained for *w* = 5, *g* = 1, *l* = 20, and *x* = 8 for 1,716 obtained clusters. As already observed in Fig1, on panel A, the highest value of the *C* measure was obtained for the lowest value of the *w* parameter. If the value of the *s* measure for a particular cluster is 1, it means that all sequences in this cluster are annotated with the same significant GO term. If the value of the *s* measure is 0.5, it means that half of the sequences in that cluster are annotated with that significant GO term. For both panels we can observe a peak for the clusters for which *s* measure value equals to 0.5. This is mostly due to a specific case of small clusters having two sequences only where one of them is annotated with a significantly enriched GO term. In general, clustering results with the lowest value of the C measure contain almost 50% of clusters in which no sequences are annotated with any significantly enriched GO term.

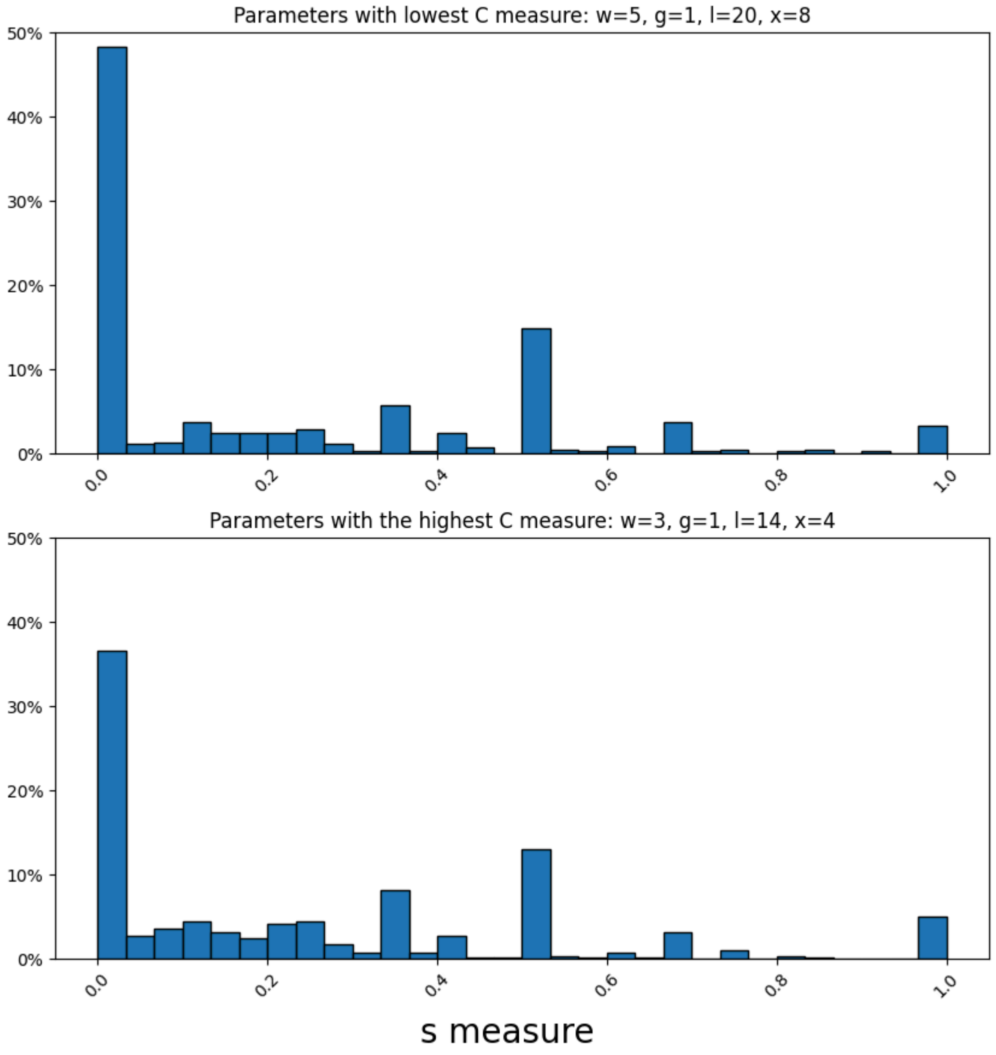

Fig S3. Histogram of the *s* measure values for the clustering results corresponding to the best and worst values of the *C* measure, expressed as a percentage of detected clusters. For the clustering results with the lowest *C* measure value (top panel), we observe that almost half of the clusters do not contain any sequences annotated with significant GO terms. The number of such clusters for the results with the highest C measure (bottom panel) is lower. Note that singleton clusters are excluded from this analysis.

Summary

In this research, we have proposed an evaluation function that can be used to select a particular set of the GBSC parameters. The function considers the number clusters with at least one significant GO term and it can be used to find the best parameter setting for the

GBSC algorithm. We showed that shorter sequences form clusters with higher *C* values, meaning that there are more clusters obtained with at least one significant GO term annotation of its sequences. We observe no dependencies between the values of the *C* measure and the sizes of the clusters.

We also showed that the parameter *w* influences the mean sequence length in the clusters (and consequently the value of the *C* measure). The parameter *g* also influences length of the sequences, but only within the groups defined by the values of the parameter *w*. As for the cluster sizes, we see that they are somehow dependent on the values of the parameter *g*. Finally we observed the relationship between the values of the parameter *x* and the values of the *C* measure but again, only within the cluster groups defined by the value of the parameter *w*.

We have also proposed the *s* measure to evaluate which of the obtained clusters contain sequences with a consistent biological function. This measure favors clusters in which the majority of sequences are annotated with the same GO term.

The code allowing to reproduce the results presented in this material and to calculate the values of the *s* and *C* measures is available at the GitHub repository: https://github.com/Addreoran/functional_evaluation_of_protein_clusters

The code for calculating the *s* measure for any set of GBSC clusters is available in the following GitHub repository:
https://github.com/agruca-polsl/gbsc_clusters_functional_analysis
