# Supplementary material for: GBSC: graph-based sequence clustering method for similar short tandem repeats in protein sequences
Source: Bioinformatics. 2026 Jun 13;42(7):btag378. doi: 10.1093/bioinformatics/btag378 (PMC13360283; doi:10.1093/bioinformatics/btag378)
Supplement: btag378_Supplementary_Data [file btag378_supplementary_data.zip › S03_clustering_parameters.pdf]

MMseqs		
Param.	Val.	Comment
min-seq-id	0.5	Sequences must have at least 50% alignment identity.
c	0.8	At least 80% of target sequence has to be included in an alignment.
cov-mode	1	Coverage of target sequence set to target sequence to allow shorter sequences join clusters with longer sequences. This is similar to how GBSC assigns sequences to clusters.
comp-bias-corr	0	Turn off to analyse low complexity regions.
mask	0	Turn off to analyse low complexity regions.
dbtype	1	Protein database.
**CD-HIT**		
Param.	Val.	Comment
b	10	Set an alignment bandwidth to 10 since STRs are short and should contain repeats throughout a sequence.
c	0.8	Sequence identity threshold.
d	0	Sets description length to the first whitespace character.
l	4	Filter out sequences with length less or equal to 4 residues.
t	0	Disable short word filtering.

Table S5. Description of MMseqs and CD-HIT parameters used for the analysis.
